# Controlled biosynthesis of gold nanoparticles with *Coffea arabica* using factorial design

**DOI:** 10.1038/s41598-019-52496-9

**Published:** 2019-11-05

**Authors:** Wanderson Juvencio Keijok, Rayssa Helena Arruda Pereira, Luis Alberto Contreras Alvarez, Adilson Ribeiro Prado, André Romero da Silva, Josimar Ribeiro, Jairo Pinto de Oliveira, Marco Cesar Cunegundes Guimarães

**Affiliations:** 1 Federal University of Espírito Santo, Department of Morphological Sciences, Vitória, 29047-10 Brazil; 2 Federal Institute of Espírito Santo, Department of chemistry, Serra, 29173-087 Brazil; 3 Federal Institute of Espírito Santo, Department of chemistry, Aracruz, 29192-733 Brazil; 4 Federal University of Espírito Santo, Department of chemistry, Vitória, 29075-910 Brazil

**Keywords:** Biochemistry, Biotechnology

## Abstract

Green synthesis of metallic nanoparticles has become incredibly popular, mainly by minimizing problems of environmental contamination and by being able to reduce, stabilize and potentially functionalize nanomaterials. Such compounds have possible applications in various areas, *e.g*., pharmaceuticals (drug delivery systems, cosmetics), textile industry (clothing with antimicrobial properties), diagnostic medicine (imaging, high efficiency biosensors), energy (solar panels), bioremediation, among others. However, the lack of reproducibility and information on the control mechanisms during synthesis have made the application of green-synthesized nanoparticles unfeasible. Thus, this study proposed the investigation of the main mechanisms affecting synthesis control, using factorial design for the preparation of gold nanoparticles with extract of *Coffea arabica*. We obtained stable (Zeta Potential, UV-vis and DLS), monodisperse, and quasi-spherical (TEM) nanoparticles, which presented adsorbed aromatic molecules (FTIR and RAMAN) and defined crystal structure (XRD), proving that the plant extract acted as a reducing agent, as well as a stabilizer and functionalizer for the synthesized nanostructures. The factorial design employed here to obtain gold nanoparticles with *Coffea arabica* extract allowed for a controlled and reproducible synthesis, enabling new possibilities for the application in several fields.

## Introduction

Metallic nanoparticles have been extensively applied in various technologies, mainly as a result of their interesting optical, electromagnetic and area/volume properties^1–4^. Besides metallic nanoparticles, other nanoparticles (such as polymeric NPs, mesoporous silicon NPs, two-dimension NPs) have also been widely investigated^5–11^. However, the effective application of these materials in nanotechnological devices requires the development of highly controlled systems that regulate size, dispersion and yield control. Different physical and chemical processes are currently used to synthesize metallic nanoparticles, allowing for the production of particles with the desired characteristics^12,13^. However, such methods are generally expensive and potentially hazardous to the environment and to living organisms^14,15^. The development of non-toxic, reliable, biologically compatible and environment-friendly processes for the synthesis of nanoparticles^16,17^ is, therefore, of broad interest. During the last decade, it has been shown that many biological systems, including plants^15,18^, algae^19^, bacteria^20,21^, fungi^22,23^ and human cells^24,25^ can transform inorganic metal ions into metal nanoparticles through the reducibility of molecules and substances present in these organisms. The production of nanoparticles using plants has advantages, such as low cultivation costs, short production time, safety and the ability to increase production volumes, making them an attractive platform for the synthesis of metallic nanoparticles^15,18^. Plants produce functional biomolecules that can actively reduce metallic ions, which stabilizes and consequently functionalizes them. In this study, we used *Coffea arabica* extract as a model due to its wide range of phenolic compounds^25^. Phytochemical analysis of arabica coffee revealed the presence of phenolic compounds and their derivatives, such as chlorogenic acids, alkaloids (caffeine), carbohydrates, lipids, volatile and heterocyclic compounds^26^. Despite the diverse applicability of these nanomaterials, the standardization of green nanoparticle synthesis remains a challenge. In particular, the influence of synthesis parameters on growth and particle size. Moreover, the great diversity of compounds involved in reduction and stabilization processes makes the achievement of reproducible synthetic processes quite impossible. Therefore, this study brings forward a detailed evaluation of the main variables that interfere in synthesis yield and control, proposing an optimization model using factorial design that will work as a model platform for controlled synthesis of nanoparticles of controlled size.

## Results and Discussion

### Complete factorial design

A number of variables (pH, time, temperature, extract concentration and agitation) were analyzed using fractional factorial design (2^5-1^) (Table 1), in order to determine which were the most significant ones (p 0.05) for the synthesis of AuNPs using full width at half maximum (FWHM), area under the curve (600 to 800 nm), maximum lambda (max λ) and maximum absorbance (Max abs) as the response variable (Table S1).

**Table 1:** Matrix of the fractional factorial design of the syntheses of 16 gold nanoparticles with *Coffea arabica* extract.

Run	Variables				
	Time (min)	Concentration (mg/mL)	pH	Temperature (°C)	Agitation (rpm)
1	20 (−1)	0,05 (−1)	2 (−1)	50 (−1)	0 (−1)
2	35 (+1)	0,05 (−1)	2 (−1)	50 (−1)	400 (+1)
3	20 (−1)	0,5 (+1)	2 (−1)	50 (−1)	0 (−1)
4	35 (+1)	0,5 (+1)	2 (−1)	50 (−1)	400 (+1)
5	20 (−1)	0,05 (−1)	9 (+1)	50 (−1)	0 (−1)
6	35 (+1)	0,05 (−1)	9 (+1)	50 (−1)	400 (+1)
7	20 (−1)	0,5 (+1)	9 (+1)	50 (−1)	0 (−1)
8	35 (+1)	0,5 (+1)	9 (+1)	50 (+1)	400 (+1)
9	20 (−1)	0,05 (−1)	2 (−1)	70 (+1)	0 (−1)
10	35 (+1)	0,05 (−1)	2 (−1)	70 (+1)	400 (+1)
11	20 (−1)	0,5 (+1)	2 (−1)	70 (+1)	0 (−1)
12	35 (+1)	0,5 (+1)	2 (−1)	70 (+1)	400 (+1)
13	20 (−1)	0,05 (−1)	9 (+1)	70 (+1)	0 (−1)
14	35 (+1)	0,05 (−1)	9 (+1)	70 (+1)	400 (+1)
15	20 (−1)	0,5 (+1)	9 (+1)	70 (+1)	0 (−1)
16	35 (+1)	0,5 (+1)	9 (+1)	70 (+1)	400 (+1)

Detailed results can be found in the supplementary material (Fig. S2). As pH and temperature were the variables that interfered the most with the response, those were chosen for the optimization of size-controlled synthesis of nanoparticles using complete factorial design (3^2^). FWHM, Max abs and Max ʎ responses were used to evaluate the influence of the synthesis on the monodispersion, reaction yield, and size of the respective nanostructures, as can be seen in Table 2.

**Table 2:** Complete factorial design results of the gold nanoparticles synthesized with coffee extract. Response variables: Max abs, FWHM and Max ʎ.

Run	Variables		Response		
	pH	Temperature (°C)	Max abs	FWHM	Max ʎ
1	3,4 (−1)	30 (−1)	0,202	2,478	556
2	5,4 (0)	30 (−1)	0,187	1,549	567
3	7,4 (+1)	30 (−1)	0,382	71,207	557
4	3,4 (−1)	50 (0)	0,550	41,506	545
5	5,4 (0)	50 (0)	0,627	61,749	533
6	7,4 (+1)	50 (0)	0,606	80,789	544
7	3,4 (−1)	70 (+1)	0,531	57,771	557
8	5,4 (0)	70 (+1)	0,602	64,829	536
9	7,4 (+1)	70 (+1)	0,562	71,624	545
10	5,4 (0)	50 (0)	0,660	61,982	533
11	5,4 (0)	50 (0)	0,609	61,898	533

To measure the statistical significance, analysis of variance (ANOVA) was performed on the best fit for the model (Table 3). ANOVA of FWHM, Max abs and Max ʎ revealed that all independent variables were significantly related with a significance level of 0.05. The methodologies that help the experimenter achieve the ideal response goal are referred to as response surface methods. These methods are used exclusively to examine the surface, or the relationship between the response and the factors affecting it. The optimal response value can be a maximum or minimum value, depending on the product or on the process in question.

**Table 3:** ANOVA of nanoparticles’ synthesis.

Maximum Absorbance	SQ*	Df*	MS*	F*	P*
(1) pH(L)	**0,010082**	**1**	**0,010082**	**7,2306**	**0,036102**
pH(Q)	0,000559	1	0,000559	0,4012	0,549816
(2) Temperature (°C) (L)	**0,141067**	**1**	**0,141067**	**101,1707**	**0,000056**
Temperature (°C) (Q)	**0,055630**	**1**	**0,055630**	**39,8966**	**0,000735**
Error	0,008366	6	0,001394		
Total SS	0,223208	10			
**FWHM**					
**(1) Temperature (°C) (L)**	**3699,668**	**1**	**3699,668**	**9,481959**	**0,021683**
Temperature (°C)(Q)	1050,804	1	1050,804	2,693128	0,151891
(2) pH(L)	448,317	1	448,317	1,149002	0,324968
pH (Q)	784,019	1	784,019	2,009379	0,206107
Error	2341,078	6	390,180		
Total SS	7943,206	10			
**Maximum lambda**					
(1) Temperature (°C)(L)	294,000	1	294,0000	5,516129	0,057164
Temperature (°C) (Q)	**470,744**	**1**	**470,7439**	**8,832258**	**0,024896**
(2) pH(L)	24,000	1	24,0000	0,450296	0,527168
pH (Q)	111,411	1	111,4105	2,090323	0,198370
Error	319,789	6	53,2982		
Total SS	1396,000	10			

*SQ – Sum of Squares; DF – Degrees of Freedom; MS – Mean Square; F – statistic test; P – p value; L – linear; Q – Quadratic. The significant variables are shown in bold.

**Figure 1:**
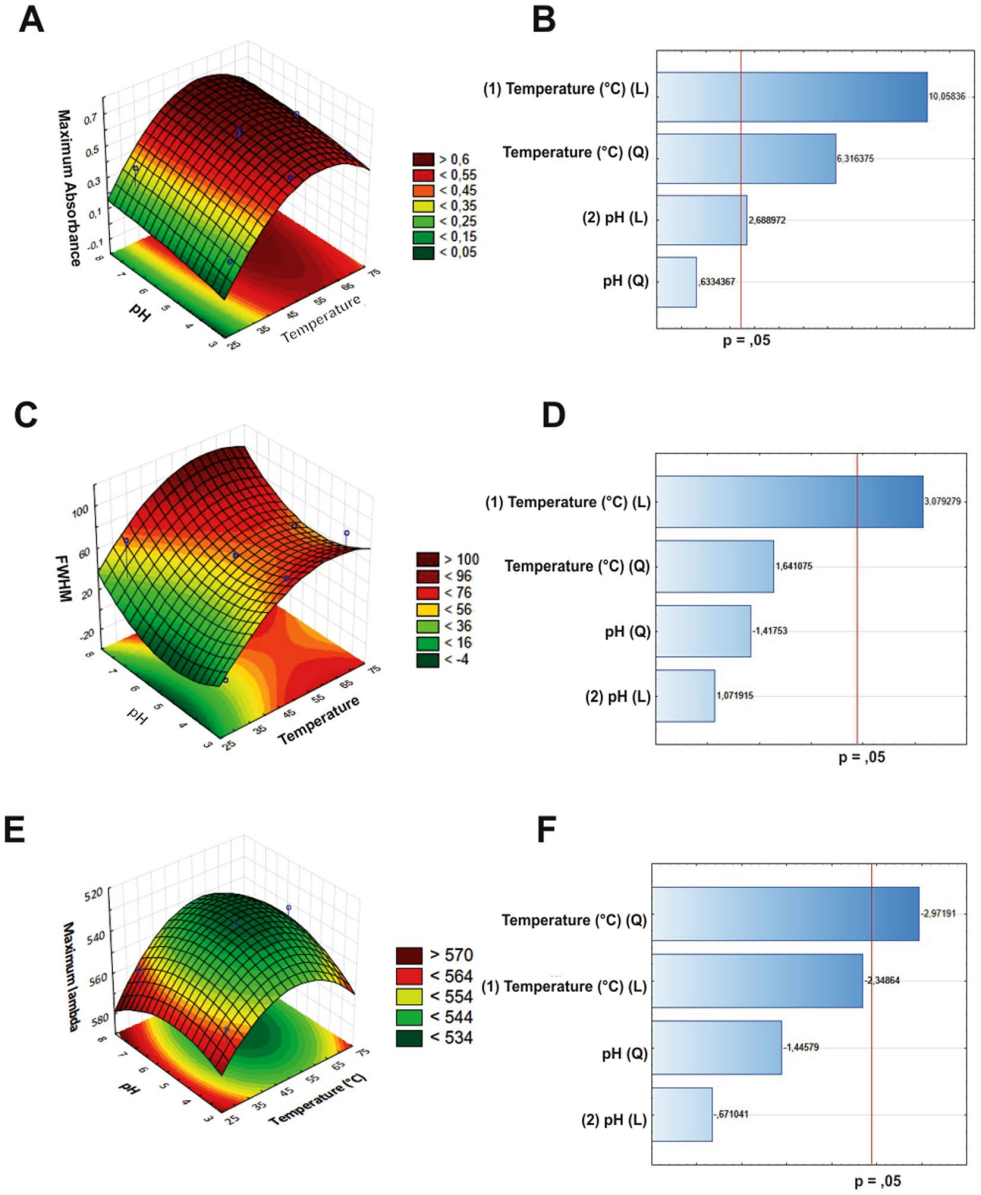
Surface response graphs of nanoparticles synthesized with coffee. Max abs (**A**), FWHM (**C**) and Max ʎ (**E**) as a function of pH and Temperature; Pareto charts indicate the variables that were significant in the process for Max abs (**B**), FWHM (**D**) and Max ʎ (**F**). Bars that exceed the vertical line (**B**,**D**,**F**) indicates that the terms are significant (p < 0.05).

Accordingly, in order to obtain optimal response, the interaction between the two variables analyzed (temperature and pH) were visualized through the response surface graphs and the Pareto charts for the complete factorial design, which revealed the influence of the variables under consideration (Fig. 1). In the Pareto chart, one can see that temperature – which crosses the reference line at 0.05 – represents the factor with the highest statistically significant influence (Fig. 1).

Rather than characterize the entire surface response, research often aims to identify conditions under which the response is optimal. To that end, an experimental strategy needs only to determine the combinations of factors that optimize the response. Therefore, the results obtained through the surface graphs made it possible to establish – both representatively (Fig. 1) and numerically (Table 4) – the best response when the maximum yield (Max abs), the nanomaterial with highest monodispersion (FWHM) and the size or shape control (Max ʎ) were analyzed as response variables.

**Table 4:** Optimal conditions, using maximum absorbance, FWHM and maximum lambda as response.

Maximum Absorbance	Values
pH	8,15838
Temperature (°C)	60,34737
**FWHM**	
pH	4,90864
Temperature (°C)	62,19245
**Maximum lambda**	
pH	5,70159
Temperature (°C)	55,13514

Maximum absorbance was chosen as the response variable for nanomaterial characterization, for this parameter is related to the yield in the formation of colloidal metallic nanoparticles.

**Figure 2:**
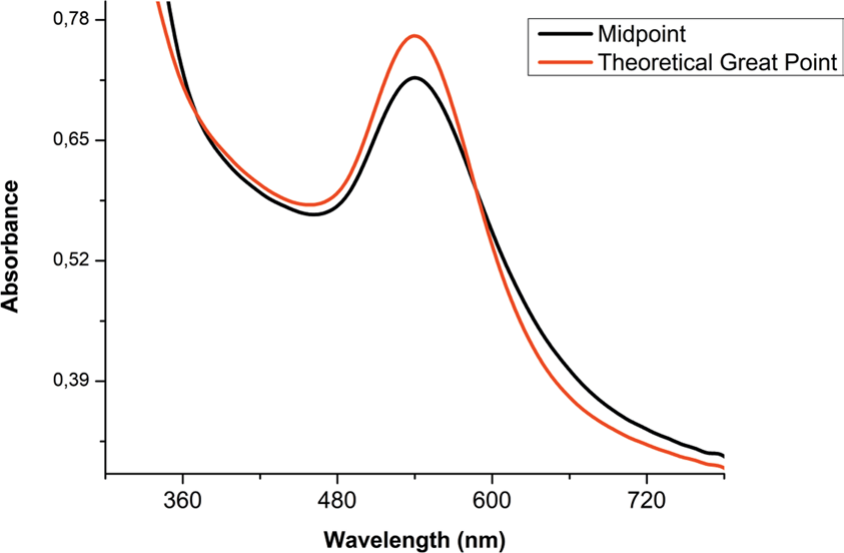
UV-Visible absorption spectroscopy of the synthesis of gold nanoparticles from the midpoint of full factorial design and the optimal point.

In order to validate the statistical model employed here, the synthesis of AuNPs was conducted with the previously defined optimal conditions for maximal absorbance, with the results being shown in the UV-Vis spectroscopy plot of Fig. 2. We verified that the predicted optimal point has higher values of maximum absorbance and lower bandwidth than the midpoint (best evaluated condition), confirming the accuracy of the model. It can also be asserted that the factorial statistical design is a useful tool for the optimization of variables that affect green synthesis.

The aim here is to find the combination and conditions associated with optimal response without having to evaluate all possible combinations of surface response factors. A complete factorial design allowed us to find the optimal pH and temperature combination to obtain the best response during the experimental treatment (Table 5).

**Table 5:** Comparison between midpoint response variables and the theoretical optimal point.

Run	Response			
	FWHM	λ maximum	Abs. maximum	Area below the curve
Midpoint	71,526	540	0,148	14,620
Theoretical Great Point	66,452	540	0,182	12,834

Several points of the response were evaluated during the comparison between the midpoint of the complete factorial design and the statistically optimal point. Our results revealed that, while the optimal point leads to improvement in FWHM, maximum absorbance and area below the curve, it does not affect maximum lambda (Table 5), positively influencing the response.

### Characterization of AuNPs

**Figure 3:**
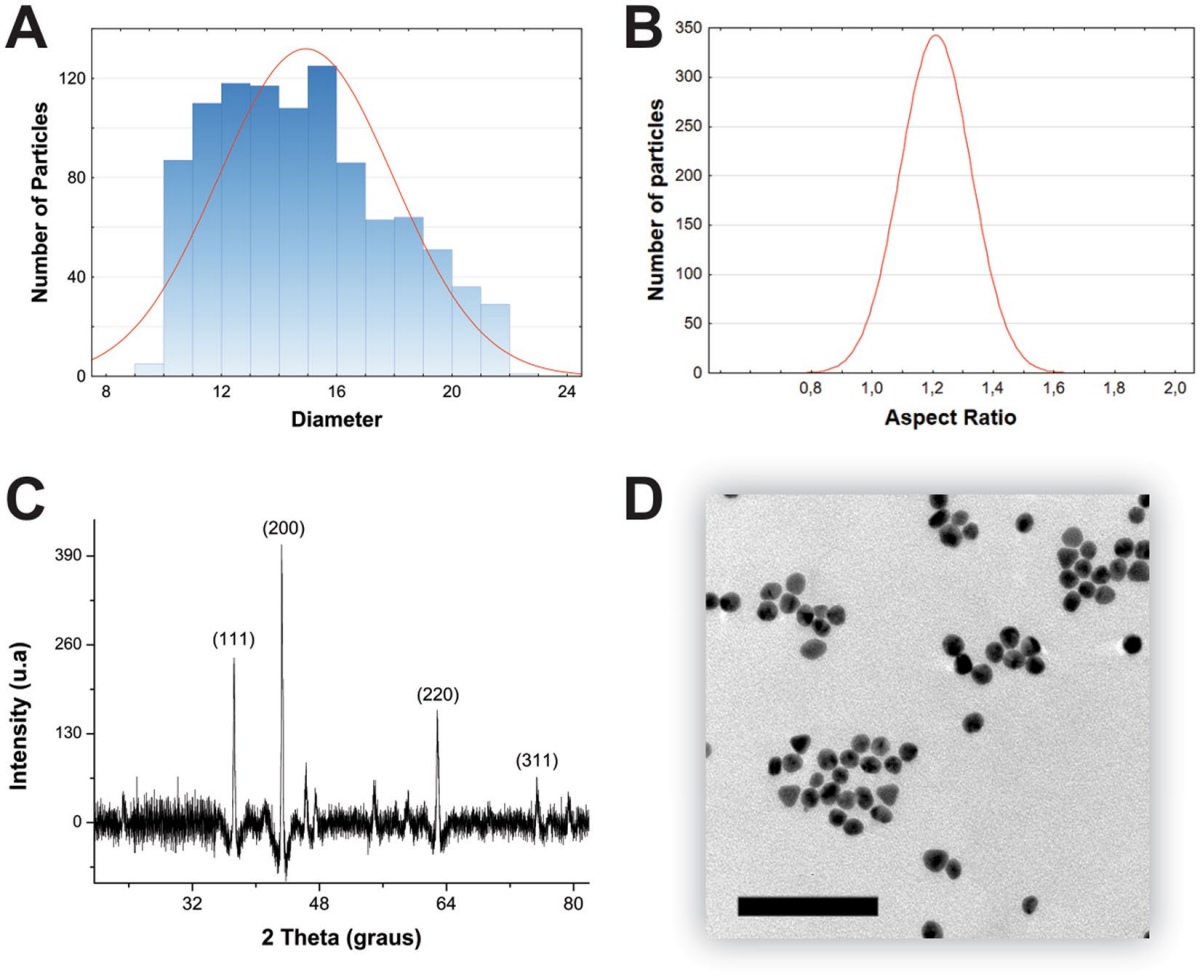
Histogram showing the Gaussian distribution of diameter (**A**) and aspect ratio of 1000 particles obtained from images acquired by transmission electron microscopy (**B**); X-ray diffractogram of AuNPs (**C**); Images obtained by transmission electron microscopy; scale bar 100 nm (**D**).

The gold nanoparticles synthesized with coffee extract had an average diameter of 14,90 ± 3,02, as determined by the counting of transmission electron microscopy (TEM) images of 1000 nanoparticles (Fig. 3A).

TEM images revealed a predominance of spherical shapes (Fig. 3D). Through the analysis of the aspect ratio (AR) data obtained from the measurement of TEM-acquired images of 1000 nanoparticles, we could verify the quasi-spherical (AR 1,17 ± 0,12) nature of the nanoparticles (Fig. 3B).

**Figure 4:**
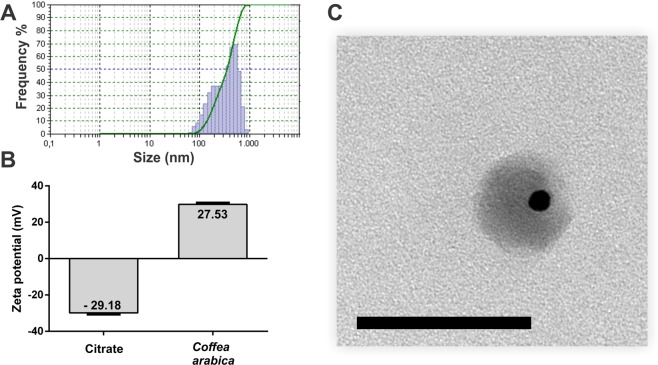
Nanoparticle DLS graph as a function of size frequency (**A**); Comparison between the zeta potential of traditional sodium citrate chemical synthesis and *Coffea arabica*-optimized green synthesis (**B**); Image obtained after the material was stained with uranyl acetate. It is possible to observe the presence of the extract around the particle, confirming the DLS response; scale bar 100 nm (**C**).

The crystalline nature of AuNPs was confirmed by XRD analysis (Fig. 3C). Four intense diffraction peaks of 38.31°, 44.45°, 64.64° and 77.73° were observed at 2Ө, corresponding to (111), (200), (220), and (311) reflections of crystalline metallic gold, respectively (Fig. 4C). A strong diffraction peak (200) suggests that this pattern reflects the predominant orientation of AuNPs. The concentration determined by ICPMS was 34.4695 mg.L^−1^ Au. The analysis of the gold nanoparticles through TEM (Fig. 3A) and dynamic light scattering (Fig. 4A) revealed different diameter scales, as a result of DLS having been employed to measure the hydrodynamic diameter of nanoparticle suspensions. The different values observed for the same nanomaterials are a consequence of the cloud formed by molecules coming from the plant extract, for the connection of these molecules with the surface of the nanomaterial results in larger hydrodynamic diameter. It is possible to visualize the extract around the particles as a result of previous negative staining with uranyl acetate (Fig. 4C), which visually confirmed the adsorption of the extract. Therefore, the difference between the average diameter obtained by TEM (approximately 14 nm) and DLS (approximately 500 nm) arises from the phenomenon of adsorption and the way it affects the hydrodynamic radius, as can be seen in Fig. 4A.

The zeta potential of AuNPs synthesized by traditional sodium citrate chemistry was also investigated and compared to green synthesis under optimal conditions with *Coffea arabica* (Fig. 4B). The analysis changed from a negative value for AuNPs with citrate (29.18 ± 1.33 mV) to a positive value for AuNPs with *Coffea arabica* (+28.33 ± 1.59 mV), implying in nanoparticles with incipient instability^27,28^. A possible explanation for the positive value of zeta potential is the adsorption of protonated biomolecules.

### Structural analysis of nanoparticle surface

The absorption assignments (Table 6) from the FTIR spectra of the coffee extract may be associated with chlorogenic acids, aromatic compounds, fatty acids and carbohydrates, among other specific components of coffee samples.

**Table 6:** FTIR spectra of Arabica coffee samples.

Bands (cm^−1^)	Assignments
1680	Stretching of the C=O bond of aldehyde or ketone aromatic compounds
1380	Stretching of the connection C=C
1265	C-H folding in the plane
1043	C-O deformation
811	C-H curvature off-plane
760	Stretching C-X (X = F, Cl, Br or I)

**Figure 5:**
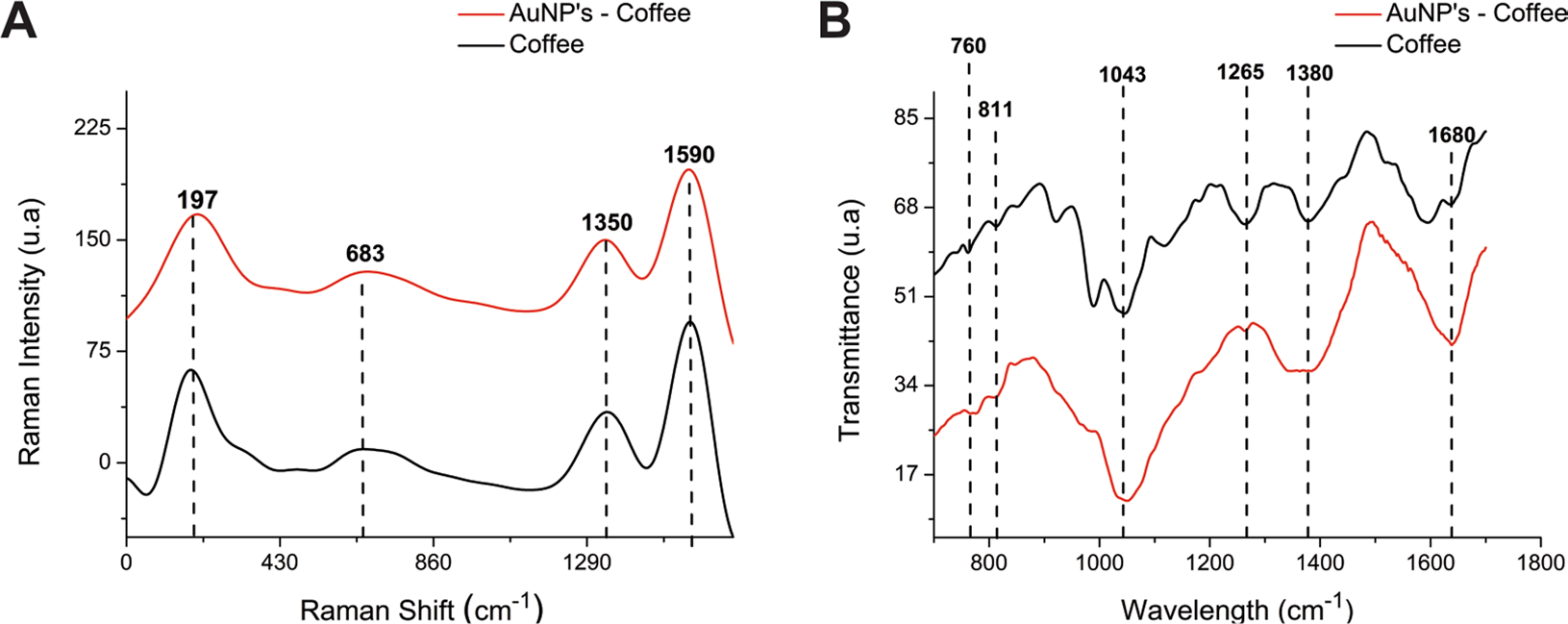
Comparison of the Raman spectra of the *Coffea arabica* extract and AuNPs (**A**). FTIR spectra of the *Coffea arabica* extract and AuNPs (**B**).

In this approach, the presence of a strong band between 1550 and 1750 cm^−1^ in the spectra can be attributed to the caffeine molecule, whereas a strong absorption band between 1150 and 1300 cm^−1^ is attributable to chlorogenic acid^18,19^ (Fig. 5A). This new profile is likely due to the oxidation-reduction process needed for Au^0^ formation, which can occur through polyphenolic molecules that reduce Au^3+^ by means of phenolic alcohols or carboxylic groups. It could also occur as a result of thermal instability of some of the extract components, such as chlorogenic acids and lactones.

The spectra produced by the Raman scattering of AuNPs and lyophilized coffee extract showed intense stretching modes with Raman displacement near 1350 and 1590 cm^−1^, corresponding to stretches of symmetrical and asymmetrical carboxylates, respectively (Fig. 5B). The band at 683 cm^−1^ may belong to caffeine, as a double medium duplet for caffeine between 643 and 741 cm^−1^ has been previously reported^21^ (Fig. 5B). Infrared spectroscopy and Raman scattering confirm that the extract components are creating a coating layer of sorts.

### Stability of AuNPs

**Figure 6:**
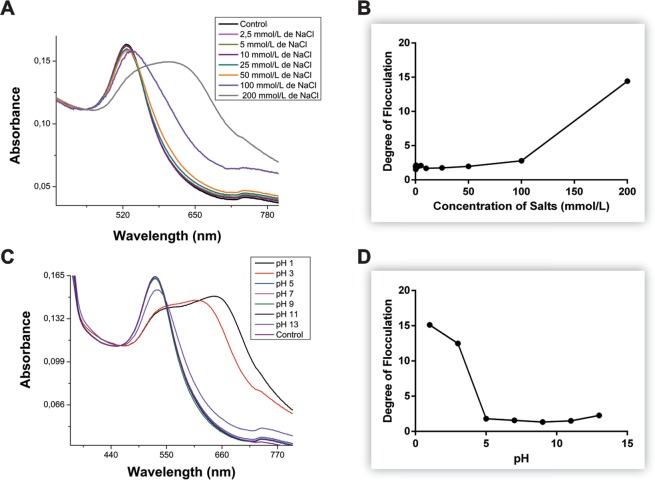
Absorption spectra showing the AuNPs colloid with coffee extract at optimized formulation, different salt concentrations (**A**) and pH (**C**). Flocculation degree based on the increase of the area between 600 and 800 nm in different salt concentrations (**B**) and pH (**D**).

To address stability related to ionic strength, the flocculation parameter was evaluated under increasing concentrations of NaCl (2.5 mM to 200 mM), as can be seen in Fig. 6A,B. In this case, one can observe that starting from 100 mM of NaCl, the absorption spectrum shows a slight shift to the right (redshift) and an expressive absorption increase in the region of 600 to 800 nm, indicating a possible loss of stability and consequent aggregation of AuNPs. To evaluate the stability in different pH ranges, the colloid was placed under different H^+^ concentrations in the medium (pH from 1 to 13)^31,32^, as shown in Fig. 6C,D. It can be observed that the pH has a direct influence on the stability of nanomaterials, given that the acidic medium (pH < 5) promoted colloid aggregation. However, the nanomaterials showed good stability over a broad pH range in acidic and basic media (pH 5 to 11). These results demonstrate that the AuNPs synthesized with *Coffea arabica* extract have good stability for a wide range of applications in biological systems.

**Figure 7:**
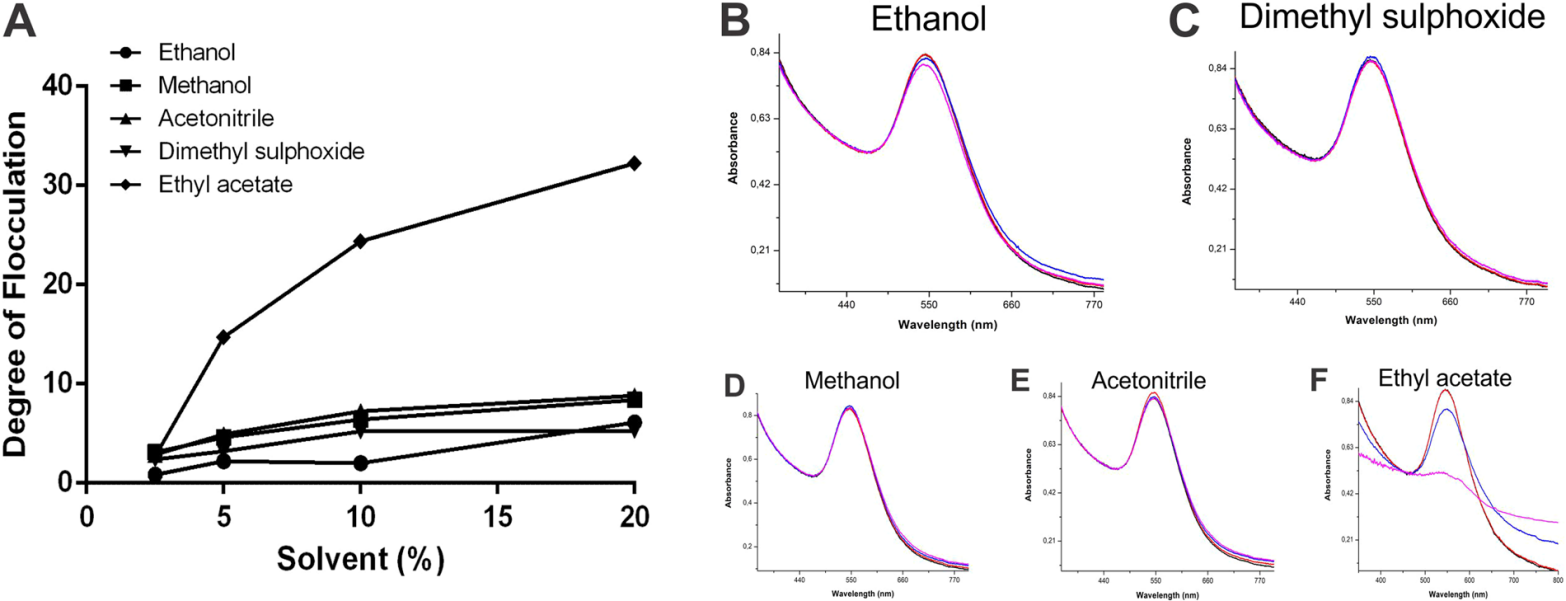
Degree of flocculation based on area increase between 600 and 800 nm at different concentrations of various solvents (**A**). Absorption spectra showing gold colloids with different solvent concentrations (2.5%, 5%, 10% and 20%): Ethanol (**B**); Dimethyl sulphoxide (**C**); Methanol (**D**); Acetonitrile (**E**) and Ethyl acetate (**F**).

The entire absorption spectrum area between 600 and 800 nm, at different concentrations of various solvents, demonstrates the stability of the synthesized AuNPs (Fig. 7A). At 2.5%, which is rather low, none of the solvents were able to induce changes in the area between the wavelengths under consideration, as demonstrated by the analysis of the degree of flocculation (Fig. 7A). At concentrations higher than 5%, very little changes were observed for almost all the solvents, with the marked exception of ethyl acetate: the increase in area from 600 to 800 nm was highly affected by the presence of this solvent (Fig. 7F), with much smaller changes having been detected at concentrations below 5%. These results can be considered in terms of solubilization, for possible biological applications of the nanomaterials in question.

## Methods

### Plant material

Internationally certified organic green *Coffee arabica* seeds were carefully washed with ultrapure water in order to remove impurities and dried at room temperature (25 °C) for 16 hours. The dried seeds were ground in a small electric mill (Di Grano Cadence, MDR302), transferred to an amber glass bottle, and placed in a refrigerator prior to the extraction process. Further details on the preparation of the vegetal extract can be found in the Supplementary Material. The aqueous extract obtained at 25 °C yielded better results regarding antioxidant capacity (Fig. S1).

### Gold nanoparticle synthesis

Gold nanoparticles were synthesized by reduction method using 7 ml of a 2.5 × 10^−4^ mol.L^−1^ HAuCl^4^ solution (Sigma-Aldrich). The remaining experimental conditions were defined according to the experimental design. After each synthesis step, the material was centrifuged at 8609 g for 20 minutes (MiniSpin 5418, Eppendorf). The supernatant was discarded and the pellet was resuspended in ultrapure water.

### Experimental design

An experimental design was conducted to optimize gold nanoparticle synthesis. As the number of variables affecting the synthesis are many, we chose the main levels for optimization based on a fractional factorial design. We used fractional factorial design 2^5-1^ to select significant variables, analyzing full width at half maximum (FWHM); Maximum lambda (Max ʎ); Area below curve; and Maximum absorbance (Max abs) as the response variable (Table S1). After defining the most significant variables, two of those were chosen for optimization. Thus, a complete experimental factorial design (3^2^) with three levels and two factors was performed in order to optimize the synthesis.

### Gold nanoparticle characterization

Optical properties of the colloids were evaluated by the UV-vis absorption spectrophotometer (Evolution§R 300 ThermoScientific). Mean diameter and aspect ratio of 1000 particles were analyzed from microscopy images using Image J software (TEM-JEM-1400, JEOL, USA inc. operated at 120kv). X-ray diffraction was analyzed using the Phillips PW 1710 diffractometer (Cuk radiation). Stability (Zeta Potential) and hydrodynamic size (DLS) of the nanomaterial were evaluated by the particle analyzer Microtac Zetatrac. Infrared Spectroscopy and Raman Scattering measurements were performed in FTIR (FT-MIR FTLA 2000 Bomem) and Raman (ALPHA 300 R Confocal Raman Spectrometer) modes, respectively. The total concentration of nanoparticles was determined using plasma inductively coupled to a Perkin Elmer mass spectrometer (ICP-OES) model Optima 7000, USA.

The stability of synthesized AuNPs was analyzed using the flocculation parameter, which describes a semi-quantitative measure of aggregation using the entire absorption spectrum between 600 and 800 nm, having been defined by Weisbecker^29^ and later modified by Maya^30^. Changes in colloid absorption were assessed in different pH ranges and ionic strength (NaCl), as well as different concentrations of various solvents, for in biological applications the stability of nanomaterials can be influenced by the constant changes of these conditions in the medium. Organic solvents: ethanol, methanol, dimethyl sulfoxide (DMSO), acetonitrile and ethyl acetate were of PA grade. To investigate the effect of the solvents on the flocculation degree at different ratios of solvent: water (%), the nanomaterial was centrifuged (8609 g, 20 min) and the pellet was resuspended in each respective solvent. Following a 1-hour reaction on the orbital shaker (200 rpm), UV-vis spectroscopic measurements were taken and analyzed.

## Conclusions

This study was conducted in order to make the synthesis of gold nanoparticles a controlled and reproducible process. To that end, several conditions for obtaining the extract were carried out as a function of antioxidant activity. In addition, a fractional factorial design (5-1) was performed to evaluate the variables that interfere with the yield of green gold nanoparticle synthesis using *Coffea arabica* extract.

The process was optimized through complete factorial design (3^2^) with the probing of output variables, namely: monodispersion (FWHM), synthesis yield (Max abs) and NPs final size (Max ʎ). The fitness of the model was duly verified and predicted values were confirmed after a test run.

The synthesized nanomaterials were monodisperse, quasi-spherical (RA~1.17) and showed good stability (+28 mV). In addition, it was possible to attest the adsorption of the *Coffea arabica* extract on the surface of AuNPs, using different techniques such as TEM, DLS, FTIR and Raman. Finally, an evaluation on the flocculation parameter was performed, aiming at the application of these NPs in biological systems. The NPs were found to be stable over a wide range of pH (5 to 11) and ionic strength (0.05 to 100 mmols.L^−1^ NaCl). A detailed study on the stability in the presence of different solvents at different concentrations (2.5 to 25%) showed that the synthesized nanomaterials remained stable in methanol, ethanol, DMSO and acetonitrile. However, under ethyl acetate at 5 to 25%, alterations in the flocculation parameter revealed considerable instability.

## Supplementary information

Supporting Information
