# Supplementary material for: Controlled biosynthesis of gold nanoparticles with Coffea arabica using factorial design
Source: Sci Rep. 2019 Nov 5;9:16019. doi: 10.1038/s41598-019-52496-9 (PMC6831671; doi:10.1038/s41598-019-52496-9)
Supplement: Supplementary file 1 — Supporting Information [file 41598_2019_52496_MOESM1_ESM.pdf]

Supporting Information

Controlled biosynthesis of gold nanoparticles with *Coffea arabica* using factorial design.

Wanderson Juvencio Keijok[1, +], Rayssa Helena Arruda Pereira[1, +], Luis Alberto Contreras[1], Adilson Ribeiro Prado[2], Andre´ Romero da Silva[3], Josimar Ribeiro[4], Jairo Pinto de Oliveira[1], and Marco Cesar Cunegundes Guimarães[1, *]

1Federal University of Espírito Santo, Department of Morphological Sciences, Vito´ria, 29047-10, Brazil

2Federal Institute of Espírito Santo, Department of chemistry, Serra, 29173-087, Brazil

3Federal Institute of Espírito Santo, Department of chemistry, Aracruz, 29192-733, Brazil

4Federal University of Espírito Santo, Department of chemistry, Vito´ria, 29075-910, Brazil

+these authors contributed equally to this work

*marco.guimaraes@ufes.br

Preparation of Plant Extract

Coffea arabica seeds were collected in the state of Espírito Santo, municipality of Domingos Martins, at a private property (Fazenda de Pedra Azul), during the summer (April) of 2016.

Green *Coffea arabica* seeds were carefully washed with ultrapure water to remove impurities, being then dried at room temperature 25°C for 16 hours. The dried seeds were ground in a small electric mill (Di Grano Cadence, MDR302), transferred to an amber glass bottle and placed in a refrigerator prior to the extraction process.

Aqueous and ethanolic extractions

The aqueous extract of the dried seeds was obtained with ultrapure water. About 10 grams of crushed seeds was mixed with 100 mL of ultrapure water. The aqueous extract was warmed at 25° C, 45° C, 65° C and 85° C, with four extracts being prepared with the respective temperatures. Each mixture was heated for 1 hour and kept under orbital shaking (400 rpm) at the round bottom flask coupled with *Graham* condenser. After the end of the process, the solution was filtered with a qualitative 3 µm paper and 0.22 µm cellulose fiber. The filter with retained material was placed in the oven for drying, the filter paper was reweighed after the material was dried, and the filtrate was frozen at -80° C, being removed and lyophilized (Liotop® L101 Lyophilizer) after 24 hours.

Ethanolic extracts were also prepared with crushed seeds, with heating at 25° C, 45°C, 65°C and 85°C, with four extracts being prepared with the respective temperatures. The ethanolic extracts were prepared following the same protocol employed for aqueous extraction with the exception of drying, which was conducted with the use of the oven.

Antioxidant activity of aqueous and ethanolic extracts

In order to select the extract with highest antioxidant activity, the ABTS assay was employed. Our ABTS results show that the extractive method and the temperature had a direct influence on the antioxidant activities of the extracts. However, one should consider that many substances are thermolabile, thus justifying the loss of their antioxidant activity generated by heating, which may lead to irreversible structures at high temperatures, accounting for the best yield of the aqueous extract having been obtained at 25°C.

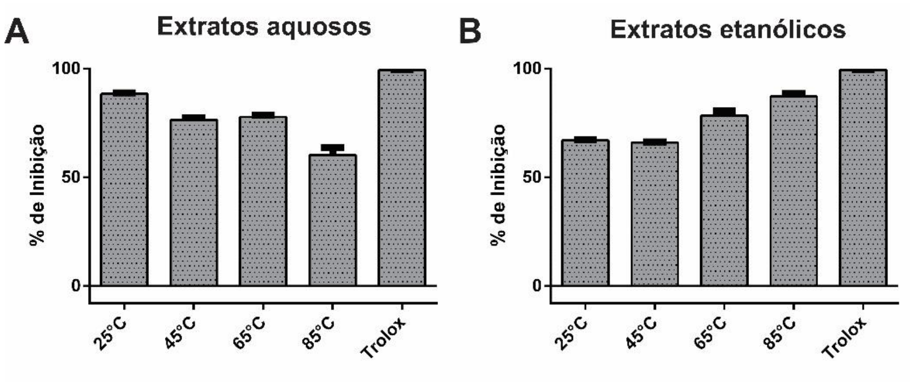

Figure S1. ABTS of aqueous (A) and ethanolic (B) extracts and their respective temperatures. Abts put trolox

FWHM

A

VARIÁVEIS	SS	DF	MS	F	P
TEMPO (min)	33,03611	1	33,03611	0,0317	0,862242
TEMPERATURA (°C)	334,888	1	334,888	0,321346	0,583299
pH	1976,102	1	1976,102	1,896196	0,198546
CONCENTRAÇÃO (mg/mL)	166,0634	1	166,0634	0,159348	0,698149
AGITAÇÃO (RPM)	53,8549	1	53,8549	0,051677	0,82475
ERROR	10421,4	10	1042,14		
TOTAL SS	12985,35	15			

B

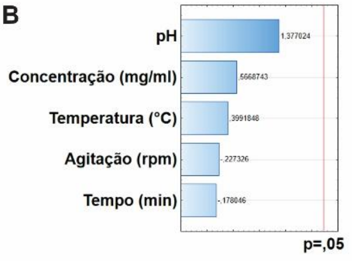

Δ λ

C

VARIÁVEIS	SS	DF	MS	F	P
TEMPO (min)	612,5625	1	612,5625	0,744927	0,40831
TEMPERATURA (°C)	473,0625	1	473,0625	0,575283	0,465667
pH	473,0625	1	473,0625	0,575283	0,465667
CONCENTRAÇÃO (mg/mL)	264,0625	1	264,0625	0,321122	0,583428
AGITAÇÃO (RPM)	22,5625	1	22,5625	0,027438	0,871738
ERROR	8223,125	10	822,3125		
TOTAL SS	10068,44	15			

D

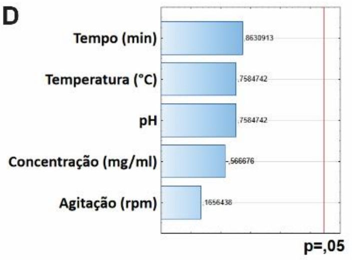

Área abaixo da curva

E

VARIÁVEIS	SS	DF	MS	F	P
TEMPO (min)	613491	1	613490,7	0,999918	0,340912
TEMPERATURA (°C)	710735	1	710735,2	1,158415	0,307075
pH	509209	1	509209,0	0,829951	0,383721
CONCENTRAÇÃO (mg/mL)	657758	1	657757,7	1,072068	0,324868
AGITAÇÃO (RPM)	638025	1	638025,3	1,039907	0,331880
ERROR	6135410	10	613541,0		
TOTAL SS	9264628	15			

F

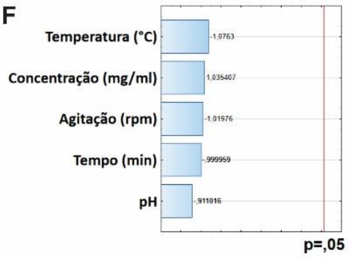

Abs. máxima

G

VARIÁVEIS	SS	DF	MS	F	P
TEMPO (min)	0,000504	1	0,000504	0,022778	0,883036
TEMPERATURA (°C)	0,027861	1	0,027861	1,260384	0,28781
pH	0,48818	1	0,48818	22,08453	0,000842
CONCENTRAÇÃO (mg/mL)	0,008653	1	0,008653	0,391463	0,545545
AGITAÇÃO (RPM)	0,001876	1	0,001876	0,084877	0,776747
ERROR	0,221051	10	0,022105		
TOTAL SS	0,748124	15			

H

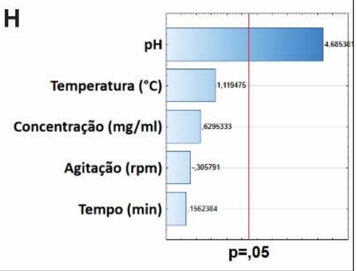

Table S1. ANOVA and Pareto chart for synthesis of gold nanoparticles using fractional factorial design 2^{5-1} analyzed in comparison to the responses of: full width at half maximum (FWHM) (A, B); Delta lambda (Δ λ) (C, D); Area below curve (E, F); Maximum Absorbance (G, H).

* **Significant variables are shown in red**; SS – Sum of squares; DF – Degree of Freedom; MSQ – Squares of Averages; F – Test F; P – p-value.
